# Primary testicular T-lymphoblastic lymphoma in a child: A case report

**DOI:** 10.1097/MD.0000000000020861

**Published:** 2020-06-26

**Authors:** Yongren Wang, Jian Li, Yongjun Fang

**Affiliations:** a Department of Hematology and Oncology, Children's Hospital of Nanjing Medical University; b Key Laboratory of Hematology, Nanjing Medical University, Nanjing, China.

**Keywords:** children, testicular, T-lymphoblastic leukemia/lymphoma

## Abstract

**Rationale:**

Primary non-Hodgkin lymphoma (NHL) of the testes is rare, representing about 9% of testicular neoplasms and 1% to 2% of non-Hodgkin lymphomas.

**Patient concerns:**

A previously healthy 47-month-old boy came to our institution for 3 months unilateral testicular swelling without tenderness. After preliminary examination, inguinal orchiectomy was performed to resect the right scrotal mass. The histopathological diagnosis of high-grade lymphoma was rendered and paraffin blocks were sent for immunophenotyping.

**Diagnosis:**

The final diagnosis by histopathological combined with immunohistochemical staining revealed primary testicular T-cell lymphoblastic lymphoma (St Jude Children's Research Hospital Staging System, stage I).

**Interventions:**

The patient was treated with right inguinal orchidectomy followed by chemotherapy (SMCC-2011 protocol modified based on the BFM-90/95 regimen from Germany) without prophylactic radiotherapy to the contralateral testis.

**Outcomes:**

After 36 months of follow-up, the patient is now disease-free without any complication.

**Lessons:**

T-lymphoblastic lymphoma should be considered in the differential diagnosis of testicular masses in children. Intensive chemotherapy may improve the prognosis of such patients.

## 1 Introduction

Primary non-Hodgkin lymphoma (NHL) of the testes is rare, representing about 9% of testicular neoplasms and 1% to 2% of non-Hodgkin-lymphomas.^[1]^ This disease occurs primarily in men over 50 years old. Diffuse large B-cell lymphoma (DLBCL) is the most common histotype of primary testicular lymphoma. However, the rare subtypes, though accounting for a minority of all lymphoma cases, are clinically important and must be recognized.^[2]^ There have been a few reports describing Burkitt/Burkitt-like lymphoma, follicular lymphoma (FL), DLBCL, and pre-B lymphoblastic lymphoma.^[3–6]^ But NHL involving testes at initial diagnosis is extremely rare in children. Herein, we report a unique case with primary testicular T-lymphoblastic lymphoma in a toddler.

## 2 Case report

**Figure 1:**
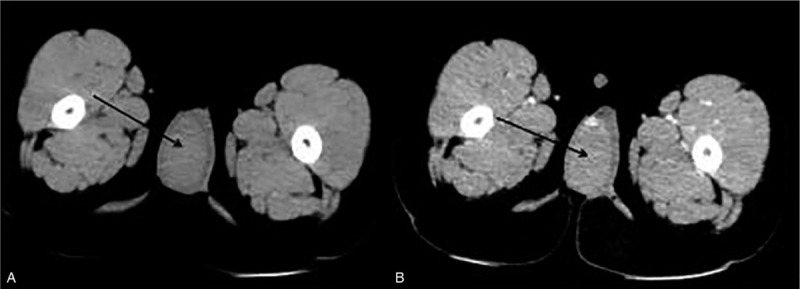
Mass in the right testis by enhanced abdomen CT scan (A: CT scan, B: CT enhanced scan). CT = computed tomography.

A 47-month-old boy presented with right testicular swelling in a local hospital in April 2016. He had no history of trauma, fever, or other complaints. His grandfather was recorded to have a history of acute leukemia. Physical examination showed unilateral enlargement of the right testis without any superficial lymph node enlargement. Ultrasound revealed asymmetrically enlarged unilateral testicle with increased vascularity but no focal mass was found. An enhanced abdominal computed tomography (CT) scan revealed a 4 × 3 × 3 cm testicular tumor that enhanced with contrast (Fig. 1). The chest CT scan was normal. Serum levels of tumor markers were within normal limits. Tests of EBV/CMV-DNA and HIV displayed negative results.

**Figure 2:**
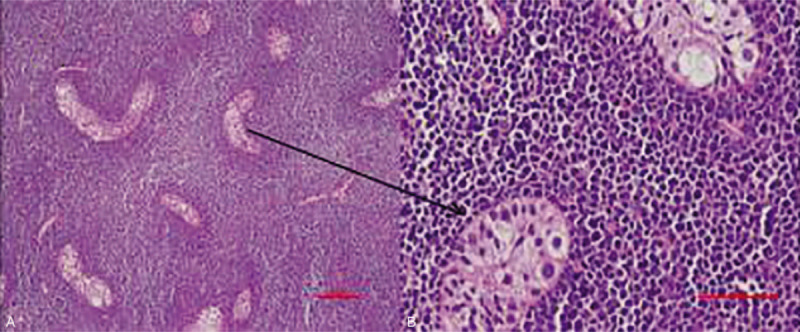
Section from testis shows diffuse neoplastic infiltrate composed of round to oval cells with irregular shaped nuclei and indistinct nucleoli (A, B) (H&E). H&E = hematoxylin and eosin.

**Figure 3:**
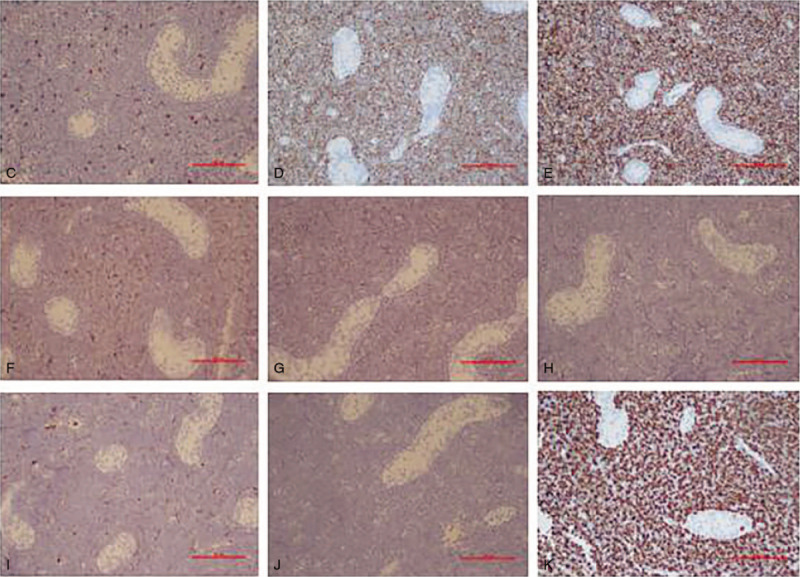
Immunophenotype of testis tissue. (C, D, and E) positivity for CD3, TdT, and CD43. F and G, Positive staining for BCL-2 and BCL-6. H, I, and J, Negativity for CD20, CD34, and MPO. K, About 80% of the neoplastic cells display nuclear Ki67 staining.

The patient received high right inguinal orchidectomy. The histopathological diagnosis of high-grade lymphoma was rendered and paraffin blocks were sent to our hospital for immunophenotyping. Hematoxylin and eosin stained sections showed a neoplastic infiltrate with an intertubular growth pattern, which was composed of small-to-intermediate sized cells with scant cytoplasm, irregular nuclei, and inconspicuous nucleoli (Fig. 2). Numerous mitotic figures and focal sclerosis were also noted. The differential diagnosis of high-grade lymphoma and leukemia was considered. The possibilities included lymphoblastic lymphoma, Burkitt lymphoma (BL), and granulocytic sarcoma. Immunohistochemistry (IHC) revealed that the neoplastic cells expressed CD43, CD3, CD99, and terminal deoxynucleotidyl transferase (TdT) and were negative for CD20, MPO, CD34, PAX5, NSE, and Desmin. Ki67 was positive in > 80% of tumor cells (Fig. 3). The patient was diagnosed with T-lymphoblastic leukemia/lymphoma and admitted to our hospital.

The peripheral blood and biochemical parameters (liver and renal function and serum lactate dehydrogenase level) were within normal limits. Bone marrow (BM) smear and biopsy did not show evidence of involvement by lymphoblastic cells. Nor did cerebrospinal fluid analysis reveal any lymphoblasts. No mediastinal and retroperitoneal enlarged lymph nodes were indicated by chest CT scans and abdominal ultrasound. The diagnosis was primary testicular T-lymphoblastic lymphoma (St Jude Children's Research Hospital Staging System, stage I). Unfortunately, the cytogenetics study was not conducted in this case. Considering the blood–testis barrier, we treated the patient with a high-dose, combined systemic, and intrathecal chemotherapy, followed by an intensive consolidation therapy (SMCC-2011 protocol modified based on the BFM-90/95 regimen from Germany) without prophylactic radiotherapy to the contralateral testis. The patient tolerated therapy well except for grade III to IV hematological toxicity and mild to moderate gastrointestinal symptoms (according to WHO Classification Standards for Toxicity of Chemotherapy Drugs). There was no evidence of testicular mass in ultrasound and CT examination after chemotherapy. Bone marrow and cerebrospinal fluid were also consistent negatives. Thus, complete remission was achieved. After a 36-month follow-up, the patient is now disease-free.

## 3 Discussion

T lymphoblastic leukemia/lymphoma (T-LBL) is a neoplasm of lymphoblasts committed to T-cell lineage involving the bone marrow and blood, or presenting as a tissue-based mass involving the thymus, lymph nodes, or extranodal sites. By convention, lymphoma refers to a mass lesion with no or minimal evidence of peripheral blood and BM involvement. Lymphomas usually attack *older* adults with male predominance.^[7]^ T-LBL frequently presents as a mass in the anterior mediastinum, often exhibiting rapid growth, and sometimes leading to a respiratory emergency. Testicular infiltration at the time of diagnosis is rare, especially in children.^[6]^ Herein, we report a pediatric patient diagnosed with primary testicular T-cell lymphoblastic lymphoma.

Primary testicular lymphoma is an uncommon and aggressive form of extranodal non-Hodgkin lymphoma in elderly males. It is rarely seen in young people. A unilateral painless swelling is the most common clinical presentation of testicular lymphoma. Our patient was admitted with a 3-month history of painless swelling in the right scrotum. Physical examination revealed an enlarged right testis. Lymph node, central nervous system, or bone marrow involvement was not found either at diagnosis or during the disease process.

Histologically, a large majority of primary testicular lymphomas (80%–90%) are of the diffuse large B-cell type.^[8]^ Other reported subtypes include FL, BL, mantel cell lymphoma, T-/NK cell lymphoma, plasmacytoma, and peripheral T-cell lymphoma.^[3,4,9–12]^ Primary testicular B-cell acute lymphoblastic leukemia has been reported in the previous literature.^[13]^ The literature review disclosed 5 cases of primary testicular pre-B lymphoblastic lymphoma in children/young adults^[5,13–16]^ (Table 1). Maria et al^[17]^ described a case of T-cell lymphoblastic lymphoma in a 38-year-old male presenting with a scrotal mass. To the best of our knowledge, primary testicular T-LBL in children has not been reported before.

**Table 1:**
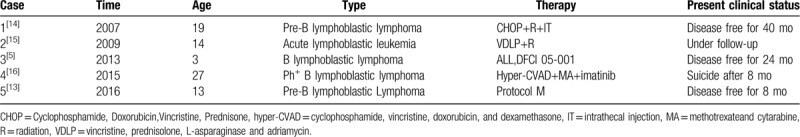
Summary of previously reported cases of primary testicular lymphoblastic lymphoma/leukemia.

The lymphoblasts in T-ALL/LBL are morphologically indistinguishable from those in B-ALL/LBL. In the smears of our case, the cells were of medium size with a high nuclear/cytoplasmic ratio, highly condensed nuclear chromatin but no evident nucleoli. Therefore, morphological examination alone could not distinguish between B- and T-cell lymphoblastic lymphoma. In this case, we used IHC for further subclassification. IHC showed the neoplastic cells expressed the TdT and CD3 antigens, which was specific for T-cells. Moreover, IHC was negative for B-cell markers (such as CD20/PAX-5) and myeloid-associated antigens (such as MPO). Further, part of the tumor cells coexpressed BCL-2 and BCL-6, which was rarely seen in primary testicular lymphoma.^[18]^

An abnormal karyotype is found in 50% to 70% of T-ALL/LBL cases. The most common recurrent cytogenetic abnormality involves the alpha and delta TCR loci at 14q11.2, the beta locus at 7q35, and the gamma locus at 7p14–15, with a variety of partners genes.^[7]^ Zhu et al^[16]^ reported a 27-year-old man with primary testicular Ph-positive B lymphoblastic lymphoma, for which fluorescent in-situ hybridization for the Philadelphia chromosome was not performed at the initial hospitalization. Unfortunately, one of the limitations of this study is the cytogenetic testing, including T-cell receptor test, was not performed.

Historically, the outcome of adult patients with PTL has been gradually improving. However, survival is still poor with intensive chemotherapy regimens, even in localized disease. Most adult PTL patients received orchidectomy followed by Rituximab—cyclophosphamide, doxorubicin, vincristine, and prednisolone, central nervous system prophylaxis and prophylactic radiotherapy to the contralateral testis with or without nodal radiotherapy, with 5-year overall survival of 85%.^[19]^ Primary testicular lymphoma in children is much rarer. Thus, treatment has not been standardized yet. In general, the prognosis of children and adolescents is much better than that of adults.^[3–5,8]^ Despite its localized presentation in our patient, primary testicular lymphoblastic lymphoma is a systemic disease. Therefore, the treatment should aim to minimize recurrence. A study on localized lymphoblastic lymphoma in children concluded that chemotherapy without radiation therapy resulted in a 30% relapse rate.^[20]^ Another study^[21]^ about primary testicular lymphoblastic leukemia/lymphoma showed a high remission rate with a median survival of ∼60 months. Recent studies^[8]^ also have demonstrated that prophylactic scrotal radiation was associated with a significant reduction in the incidence of testicular relapse and improvement in PFS and OS. However, in this case, the patient's parents refused radiotherapy. Thus, we chose a high-dose, combined systemic and intrathecal chemotherapy, followed by intensive consolidation therapy. The patient is still alive 36 months after diagnosis with no evidence of disease.

In conclusion, this case report documents the first diagnosed case of primary testicular T-lymphoblastic lymphoma (PTT-LBL) in a child and alerts clinicians and pathologists to this rare type of lymphoma at an unusual location. Moreover, intensive chemotherapy may improve the prognosis of such patients.

## Acknowledgments

The authors gratefully acknowledge the pathologists in their hospital for their expertise and generous provision of data.

## Author contributions

**Data curation:** Yongjun Fang

**Funding acquisition:** Yongjun Fang.

**Project administration:** Yongren Wang and Yongjun Fang.

**Writing – original draft:** Jian Li.

**Writing – review & editing:** Yongren Wang and Yongjun Fang.

## References

[1] Vitolo U Ferreri AJ Zucca E Primary testicular lymphoma. Crit Rev Oncol Hematol 2008;65:183–9. 17962036 10.1016/j.critrevonc.2007.08.005

[2] Gundrum JD Mathiason MA Moore DB Primary testicular diffuse large B-cell lymphoma: a population-based study on the incidence, natural history, and survival comparison with primary nodal counterpart before and after the introduction of rituximab. J Clin Oncol 2009;27:5227–32. 19770371 10.1200/JCO.2009.22.5896

[3] Koksal Y Yalcin B Uner A Primary testicular Burkitt lymphoma in a child. Pediatr Hematol Oncol 2005;22:705–9. 16251177 10.1080/08880010500278822

[4] Lones MA Raphael M McCarthy K Primary follicular lymphoma of the testis in children and adolescents. J Pediatr Hematol Oncol 2012;34:68–71. 22215099 10.1097/MPH.0b013e31820e4636 PMC3251817

[5] Garcia AV Alobeid B Traina JM Isolated primary testicular B lymphoblastic lymphoma: an unusual presentation. J Pediatr Hematol Oncol 2013;35:e88–90. 23042023 10.1097/MPH.0b013e318271c470 PMC3563753

[6] Al-Abbadi MA Hattab EM Tarawneh M Primary testicular and paratesticular lymphoma: a retrospective clinicopathologic study of 34 cases with emphasis on differential diagnosis. Arch Pathol Lab Med 2007;131:1040–6. 17616989 10.5858/2007-131-1040-PTAPLA

[7] You MJ Medeiros LJ Hsi ED T-lymphoblastic leukemia/lymphoma. Am J Clin Pathol 2015;144:411–22. 26276771 10.1309/AJCPMF03LVSBLHPJ

[8] Cheah CY Wirth A Seymour JF Primary testicular lymphoma. Blood 2014;123:486–93. 24282217 10.1182/blood-2013-10-530659

[9] Jun HJ Kim WS Yang JH Orbital infiltration as the first site of relapse of primary testicular T-cell lymphoma. Cancer Res Treat 2007;39:40–3. 19746228 10.4143/crt.2007.39.1.40 PMC2739356

[10] Takahashi M Ito K Sato K [Primary bilateral testicular plasmacytoma: a case report]. Hinyokika Kiyo 2011;57:653–6. 22166832

[11] Shi XL Xie JL Zhou XG [Primary testicular NK/T cell lymphoma: a clinicopathologic analysis of six cases]. Zhonghua Bing Li Xue Za Zhi 2018;47:168–71. 29534354 10.3760/cma.j.issn.0529-5807.2018.03.004

[12] Licci S Morelli L Covello R Primary mantle cell lymphoma of the testis. Ann Hematol 2011;90:483–4. 20714722 10.1007/s00277-010-1049-3

[13] Binesh F Yazdi MF Jenabzadeh A Primary testicular pre-B lymphoblastic lymphoma. APSP J Case Rep 2016;7:15. 27170920 PMC4852058

[14] Charandeep S Sangeeta D Primary testicular precursor B-lymphoblastic lymphoma: a rare entity. Leukemia Lymphoma 2007;48:2060–2. 17852709 10.1080/10428190701535496

[15] Biswas A Sahana PK A case of acute lymphoblastic leukemia presenting with macroorchidism in a fourteen-year-old boy: a rare presentation. Int J Hematol 2009;5:3.

[16] Zhu J Zhang S Zhu L Primary testicular Ph-positive B lymphoblastic lymphoma: an unusual presentation and review. Cancer Biol Ther 2015;16:1122–7. 26147907 10.1080/15384047.2015.1056412 PMC4622064

[17] Ambrosio MR Onorati M Rocca BJ Unusual presentation of primary T-cell lymphoblastic lymphoma: description of two cases. Diagn Pathol 2014;9:124. 24950962 10.1186/1746-1596-9-124 PMC4078934

[18] Menter T Ernst M Drachneris J Phenotype profiling of primary testicular diffuse large B-cell lymphomas. Hematol Oncol 2014;32:72–81. 23949965 10.1002/hon.2090

[19] Vitolo U Chiappella A Ferreri AJ First-line treatment for primary testicular diffuse large B-cell lymphoma with rituximab-CHOP, CNS prophylaxis, and contralateral testis irradiation: final results of an international phase II trial. J Clin Oncol 2011;29:2766–72. 21646602 10.1200/JCO.2010.31.4187

[20] Patte C Auperin A Michon J The Societe Francaise d’Oncologie Pediatrique LMB89 protocol: highly effective multiagent chemotherapy tailored to the tumor burden and initial response in 561 unselected children with B-cell lymphomas and L3 leukemia. Blood 2001;97:3370–9. 11369626 10.1182/blood.v97.11.3370

[21] Sahni C Desai S Primary testicular precursor B-lymphoblastic lymphoma: a rare entity. Leuk Lymphoma 2007;48:2060–2. 17852709 10.1080/10428190701535496

